# Association of circulating calprotectin with lipid profile in axial spondyloarthritis

**DOI:** 10.1038/s41598-018-32199-3

**Published:** 2018-09-13

**Authors:** Fernanda Genre, Javier Rueda-Gotor, Sara Remuzgo-Martínez, Alfonso Corrales, Verónica Mijares, Rosa Expósito, Cristina Mata, Virginia Portilla, Ricardo Blanco, José Luis Hernández, Javier Llorca, Oreste Gualillo, Raquel López-Mejías, Miguel A. González-Gay

**Affiliations:** 1 Epidemiology, Genetics and Atherosclerosis Research Group on Systemic Inflammatory Diseases, IDIVAL, Santander, 39011 Spain; 2 Division of Rheumatology, Hospital Comarcal de Laredo, Laredo, 39770 Spain; 3 Bone Metabolism Unit, Department of Internal Medicine, Hospital Universitario Marqués de Valdecilla, IDIVAL, University of Cantabria, RETICEF, Santander, 39008 Spain; 4 Department of Epidemiology and Computational Biology, School of Medicine, University of Cantabria, and CIBER Epidemiología y Salud Pública (CIBERESP), IDIVAL, Santander, 39011 Spain; 5 SERGAS (Servizo Galego de Saude) and IDIS (Instituto de Investigación Sanitaria de Santiago), The NEIRID Group (Neuroendocrine Interactions in Rheumatology and Inflammatory Diseases), Santiago University Clinical Hospital, Santiago de Compostela, 15706 Spain; 6 School of Medicine, University of Cantabria, Santander, 39011 Spain; 7 Cardiovascular Pathophysiology and Genomics Research Unit, School of Physiology, Faculty of Health Sciences, University of the Witwatersrand, Johannesburg, 2000 South Africa

## Abstract

Calprotectin (CPT) is released during inflammation, also in the context of atherosclerosis. The link between CPT and the atherosclerotic process was evaluated in several diseases. However, studies in axial spondyloarthritis (axSpA), associated with a high incidence of subclinical atherosclerosis, are scarce. Therefore, we assessed the association of CPT with subclinical atherosclerosis and metabolic risk factors in axSpA. CPT serum levels were measured by enzyme-linked immunosorbent assay in 163 axSpA patients and 63 controls. Subclinical atherosclerosis was determined in patients by carotid ultrasonography (assessing the presence/absence of carotid plaques and carotid intima-media thickness [cIMT]). Data on inflammation, disease activity, lipid profile and treatment were collected to evaluate its relationship with CPT. axSpA patients evidenced lower CPT levels than controls. CPT showed no association with plaques or cIMT in axSpA. CPT and HDL-cholesterol negatively correlated, while a positive association of CPT with the atherogenic index was disclosed. Additionally, axSpA patients with C-reactive protein values at diagnosis higher than 3 mg/L displayed higher CPT levels. Our study shows no relationship between CPT and markers of subclinical atherosclerosis in axSpA. Nevertheless, it demonstrates an association of CPT with adverse lipid profiles and inflammatory biomarkers, which could further influence on the development of atherosclerosis.

## Introduction

Several metabolic abnormalities such as hyperglycemia, dyslipidemia, obesity and hypertension are considered risk factors for the development of cardiovascular (CV) disease. Most of them are clustered under the term ‘Metabolic syndrome’ (MeS), a pathologic state with growing prevalence that has been associated with the development not only of vascular and cardiac diseases, but also with other pathologies^1–5^.

CV disease is generally the result of an accelerated atherosclerotic process, initiated by damage to the vascular endothelium. This can lead to structural damage, manifested by thickening of the vascular wall (carotid intima-media thickness [cIMT]) as well as atheromatous plaque formation^6^. These morphological findings are considered surrogate markers of CV disease. In this sense, both in the general population and in patients with chronic inflammatory arthritis, an abnormal cIMT value or the presence of plaques predict the development of CV events such as ischemic heart disease or stroke^7–11^. As previously shown by our group, the presence of these two surrogate markers of CV disease can be determined by carotid ultrasound, a non-invasive imaging technique^10–12^.

Patients diagnosed with chronic inflammatory diseases, such as axial spondyloarthritis (axSpA), show higher morbidity and mortality rates due to CV disease, particularly atherosclerosis, when compared to the general population^6,13,14^. This is not only the result of a higher incidence of traditional CV risk factors and MeS features^15–20^, but also due to the inflammatory burden present in these patients, that acts as an additional independent CV risk factor^21,22^. In this regard, it is well known that inflammation triggers the expression of endothelial adhesion molecules, promoting thus endothelial damage and the formation of atherosclerotic plaques, an indicator of advanced atherosclerosis^22^.

Among the cells implicated in the development of atherosclerotic disease, neutrophils, monocytes and macrophages play a key role by secreting a large number of molecules which are further involved in the inflammatory process of atherosclerosis^23^. In this context, calprotectin (CPT), also known as myeloid-related protein 8/14 (MRP8/14) or S100A8/A9, is a heterodimeric complex of proteins released by these cells during inflammation^24^. In fact, it was reported that the secretion of CPT is stimulated by the interaction between phagocytes and the endothelium^25^. Accordingly, the link between CPT and the pathophysiology of atherosclerosis has been studied in several diseases^26–29^. However, previous studies on this issue that included axSpA patients are limited to only one performed in a small cohort of patients with different inflammatory arthropaties^30^.

Regarding the functional role of CPT, this protein exerts diverse intra- and extra-cellular functions, acting on different target tissues/organs, such as muscle, cartilage, bone, synovial tissue, vasculature and epithelium, among others^31^. Modulation of the inflammatory response by binding to different cell-surface proteins such as toll-like receptor 4, as well as oxidant-scavenging, antimicrobial and apoptosis-inducing activities are among the extracellular functions attributed to CPT^31^. Interestingly, both pro- and anti-inflammatory roles have been reported for CPT^32^. Additionally, previous studies showed that CPT is a sensitive and specific biomarker of systemic and local inflammation, mirroring in the latter case intestinal or synovial inflammation (when its levels are assessed in stool samples or synovial fluid, respectively)^33–35^. Fecal CPT levels have also been associated with disease activity in ankylosing spondylitis (AS)^24,36^. Furthermore, serum CPT was shown to be an independent marker of radiographic spinal progression in axSpA^37^. Also in this line, it was reported that CPT expression is highly upregulated in inflamed axial entheses in SKG mice, an experimental model of SpA^38^.

Taking all these considerations into account, in the present study we aimed to evaluate the potential association of CPT with the development of subclinical atherosclerotic disease and also with metabolic risk factors, including lipid profile and markers of inflammation, in a large cohort of axSpA patients.

## Results

### Differences in CPT levels between axSpA patients and controls

**Figure 1:**
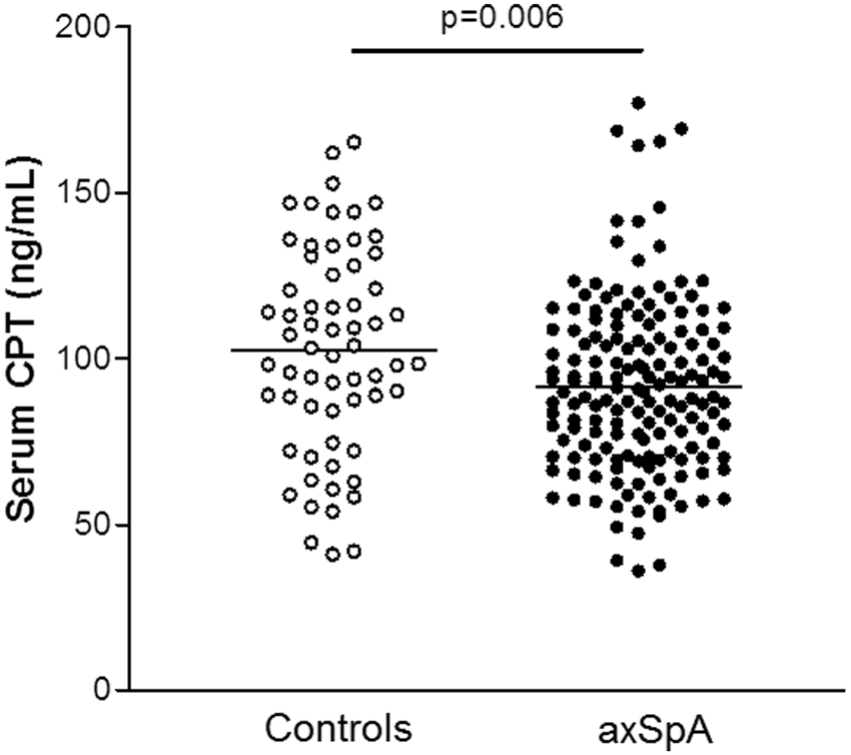
Differences in CPT serum levels between healthy controls and axSpA patients, after adjustment for sex, age at the time of the study and traditional CV risk factors (smoking, obesity, dyslipidemia and hypertension). Healthy controls (n = 63) are represented by empty circles (○), while axSpA patients (n = 163) are represented by filled circles (●). Horizontal bars indicate mean value of each group.

axSpA patients displayed statistically significantly lower CPT levels than controls (91.4 ± 26.1 vs. 102.3 ± 31.2 ng/mL, respectively, p = 0.006) after adjustment for sex, age at the time of the study and classic CV risk factors (Fig. 1, Table 1). No statistically significant differences were observed in CPT levels between AS and nr-axSpA when adjusting for sex, age at the time of the study, classic CV risk factors and HLA-B27 status (p > 0.05).

**Table 1:** Demographical, laboratory and cardiovascular disease-related data in healthy controls and patients with axSpA (both AS and nr-axSpA patients).

Variable	Controls (n = 63)	axSpA (n = 163)	AS (n = 119)	nr-axSpA (n = 44)
Men/Women, n	28/35	90/73	73/46	17/27
Age at study (years), mean ± SD	50.9 ± 15.3	43.7 ± 11.6	44.9 ± 11.9	40.3 ± 10.3
Age at axSpA diagnosis (years), mean ± SD	—	37.2 ± 10.4	36.7 ± 10.7	38.7 ± 9.2
History of classic cardiovascular risk factors, n (%)				
Current smokers	12 (19.0)	47 (28.8)	39 (32.8)	8 (18.2)
Obesity	12 (19.0)	32 (19.6)	25 (21.0)	7 (15.9)
Dyslipidemia	13 (20.6)	33 (20.2)	25 (21.0)	8 (18.2)
Hypertension	12 (19.0)	25 (15.3)	19 (16.0)	6 (13.6)
Body mass index (kg/m^2^) at study, mean ± SD	26.8 ± 4.9	25.9 ± 4.5	26.1 ± 4.6	25.2 ± 4.2
Systolic blood pressure (mm Hg) at study, mean ± SD	127.4 ± 15.4	128.1 ± 15.2	129.2 ± 15.0	125.2 ± 15.5
Diastolic blood pressure (mm Hg) at study, mean ± SD	78.2 ± 8.8	77.9 ± 10.0	78.0 ± 10.3	77.8 ± 9.2
Total cholesterol (mg/dL) at study, mean ± SD	202.2 ± 33.9	193.1 ± 35.6	194.9 ± 36.1	188.0 ± 34.1
HDL cholesterol (mg/dL) at study, mean ± SD	59.1 ± 16.0	55.4 ± 16.4	53.8 ± 14.8	59.5 ± 19.6
LDL cholesterol (mg/dL) at study, mean ± SD	122.0 ± 31.8	118.2 ± 30.0	120.7 ± 31.1	111.4 ± 25.8
Triglycerides (mg/dL) at study, mean ± SD	94.7 ± 47.7	97.5 ± 54.5	98.7 ± 54.4	94.3 ± 55.3
Atherogenic index (total cholesterol/HDL), mean ± SD	3.6 ± 1.1	3.7 ± 1.0	3.8 ± 1.0	3.4 ± 0.9
CRP (mg/L) at study, mean ± SD	2.9 ± 4.4	5.7 ± 9.9	6.3 ± 10.9	4.0 ± 5.8
CRP (mg/L) at axSpA diagnosis, mean ± SD	—	11.5 ± 23.4	14.0 ± 26.6	4.9 ± 7.4
ESR (mm/1st hour) at study, mean ± SD	11.5 ± 4.4	11.3 ± 12.4	12.3 ± 13.3	8.4 ± 8.9
CRP at axSpA diagnosis ≥ 3 mg/L, n (%)	—	86 (52.8)	71 (59.7)	15 (34.1)
ESR (mm/1^st^ hour) at axSpA diagnosis, mean ± SD	—	15.1 ± 17.9	17.2 ± 20.2	10.3 ± 10.2
BASDAI at study, median [IQ range]	—	3.80 [1.75–5.50]	3.70 [1.65–4.95]	4.40 [2.60–6.10]
BASDAI at study, mean ± SD	—	3.80 ± 2.22	3.60 ± 2.17	4.35 ± 2.29
Therapy at study				
Anti-TNF-α treatment, n (%)	—	49 (30.1)	43 (36.1)	6 (13.6)
DMARDs, n (%)*	—	79 (48.5)	62 (52.1)	17 (38.6)
NSAIDs, n (%)**	—	158 (96.9)	114 (95.8)	44 (100)
Statins, n (%)	—	13/163 (8.0)	10/119 (8.4)	3/44 (6.8)
Carotid IMT (mm), mean ± SD	—	0.608 ± 0.131	0.622 ± 0.14	0.568 ± 0.11
Carotid plaques, n (%)	—	51 (31.3)	43 (36.1)	8 (18.2)
CPT (ng/mL)	102.3 ± 31.2	91.4 ± 26.1	91.4 ± 26.7	91.1 ± 24.9

AS: Ankylosing spondylitis; axSpA: Axial spondyloarthritis; BASDAI: Bath Ankylosing Spondylitis Disease Activity Index; CPT: Calprotectin; CRP: C-Reactive Protein; DMARDs: Disease Modifying Anti-Rheumatic Drugs; ESR: Erythrocyte Sedimentation Rate; HDL: High Density Lipoprotein; IMT: Intima-Media Thickness; IQ: Interquartile; LDL: Low Density Lipoprotein; nr-axSpA: non-radiographic axial spondyloarthritis; NSAIDs: Nonsteroidal anti-inflammatory drugs; SD: Standard Deviation; TNF: Tumour necrosis factor. ^*^Mainly sulphasalazine, median dose: 2 grams/day. ^**^Mainly naproxen, median dose: 1000 milligrams/day.

### Association of CPT levels and markers of subclinical atherosclerosis

When we assessed the potential association of CPT levels with presence of plaques and cIMT values in axSpA, no statistically significant results were obtained after adjusting for sex, age at the time of the study, classic CV risk factors and HLA-B27 status (p > 0.05).

### Relationship of CPT levels with routine laboratory markers of inflammation, lipid profile, disease activity and treatment

**Figure 2:**
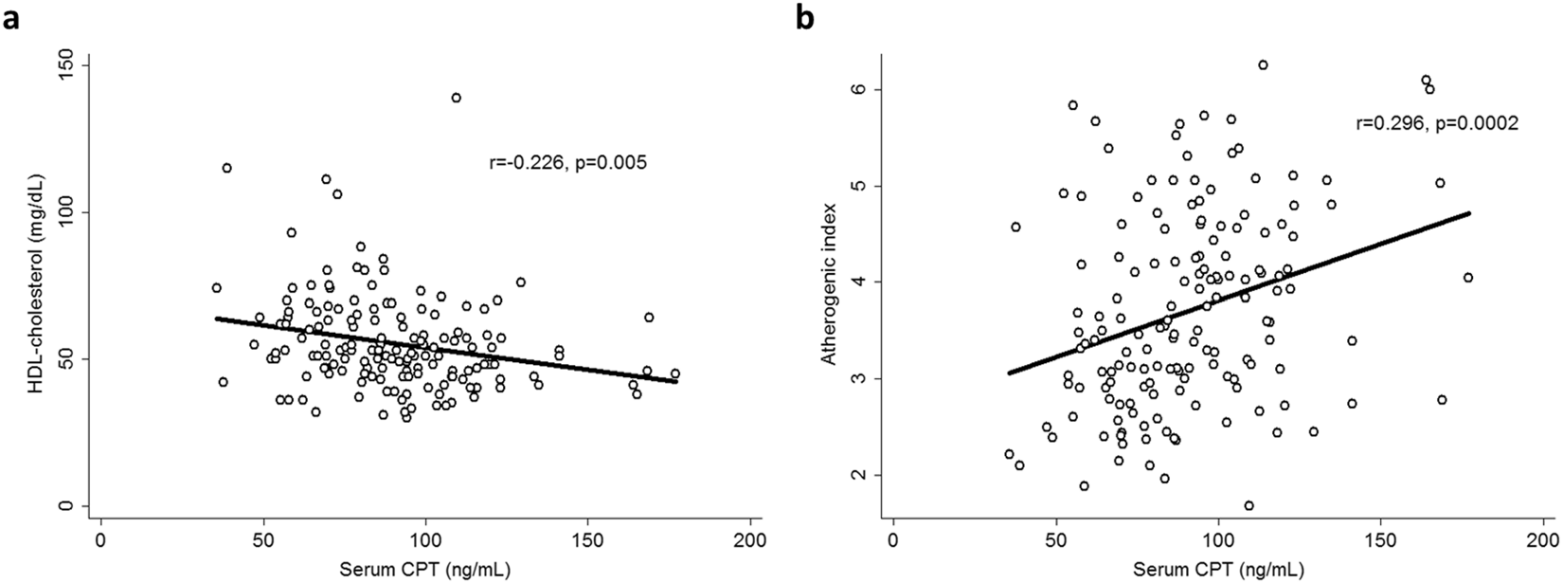
Association of serum levels of CPT with HDL-cholesterol (**a**) and atherogenic index (**b**) in axSpA patients after adjustment for sex, age at the time of the study, classic CV risk factors and HLA-B27 status.

We observed a negative correlation of CPT with high density lipoprotein (HDL)-cholesterol (r = −0.226, p = 0.005, Fig. 2a) and a positive correlation with the atherogenic index (AI) (r = 0.296, p = 0.0002, Fig. 2b) in axSpA. This association between CPT and parameters of lipid profile was further confirmed in the AS subgroup (r = −0.285, p = 0.003 for HDL-cholesterol, and r = 0.285, p = 0.003 for AI) and in the non-radiographic axSpA (nr-axSpA) subgroup (r = 0.371, p = 0.02 for AI). In addition, we found an association with C-reactive protein (CRP) levels at diagnosis in axSpA patients (r = 0.169, p = 0.03). When patients were stratified according to the subtype of axSpA, it was observed that this association was due to the AS subgroup (r = 0.188, p = 0.05). Accordingly, the mean CPT concentration in axSpA patients with CRP levels at diagnosis higher than 3 mg/L was more elevated than that found in patients with lower CRP levels at diagnosis (95.5 ± 26.4 vs. 86.8 ± 25.2 ng/mL, respectively, p = 0.01). This significant difference in CPT levels among patients with high/low CRP levels at diagnosis was also observed in AS (p = 0.001). No statistically significant association was disclosed between CPT levels and disease activity (assessed by Bath Ankylosing Spondylitis Disease Activity Index [BASDAI]) or markers of inflammation (CRP and erythrocyte sedimentation rate [ESR]) at the time of the study, or with ESR at the time of axSpA diagnosis (p > 0.05). All these results were adjusted for sex, age at the time of the study, classic CV risk factors and HLA-B27 status as potential confounding factors.

The different treatments that our axSpA patients were receiving at the time of the study (anti- tumour necrosis factor-α [anti-TNF-α] therapy, disease modifying anti-rheumatic drugs [DMARDs], nonsteroidal anti-inflammatory drugs [NSAIDs] and statins) were not found to exert any influence on their CPT serum levels after adjustment for sex, age at the time of the study, classic CV risk factors and HLA-B27 status (p > 0.05).

When we evaluated the association of CPT and lipid profile parameters in healthy controls, we observed a statistically significant correlation with HDL-cholesterol and AI after adjustment for sex, age at the time of the study and classic CV risk factors (r = −0.376; p = 0.004 for HLD-cholesterol, and r = 0.393; p = 0.003 for AI). No statistically significant association of CPT was noted regarding CRP and ESR levels at study in the controls group after adjustment for those potential confounding factors (p > 0.05).

## Discussion

CV disease is one of the leading causes of death in axSpA patients^39^. In this regard, the chronic inflammatory state as well as the higher incidence of traditional CV risk factors and MeS features in these patients play a key role in the development of CV disease in axSpA^15–20^. The elucidation of the molecular mechanisms that are implicated in the onset and progression of CV disease in axSpA could help to a better understanding of this comorbidity. Thereby, serum biomarkers could be used as an alternative, non-invasive approach to assess CV risk in axSpA patients, combined with imaging techniques such as carotid ultrasound. Thereupon, during the last years, much effort has been made to identify circulating biomarkers of CV risk in different chronic inflammatory diseases such as AS. Among them, adipokines, MeS-related molecules, as well as biomarkers of endothelial cell activation and inflammation have been reported to exert relevant roles in the development of CV disease^40^. However, given the complex physiological effects of these molecules, the identification of the ‘perfect’ biomarker of CV risk in chronic inflammatory diseases for its use in the daily clinical practice has not been achieved yet. Consequently, data on this issue are still inconclusive and require further studies.

This prompted us to assess whether CPT, a protein released during inflammation and potentially linked to CV disease in inflammatory pathologies such as type 2 diabetes mellitus or Sjögren’s syndrome^26–29^, could be associated with subclinical atherosclerosis and metabolic risk factors in axSpA.

Current data on CPT levels in patients with axSpA are very heterogeneous. Even if most of the studies report higher circulating levels of this protein in these patients^41–44^, others do not find differences as compared to controls^24,36^. In this study, that included a large cohort of patients with axSpA, we disclosed significantly lower CPT serum concentrations in our patients when compared to controls. In this regard, a recent study revealed that CPT levels decrease after the end of a 6-month intensive exercise programme in AS and nr-axSpA patients, accompanied by clinical improvement^45^. This may be particularly relevant, given that regular exercise and physical therapy is recommended to complement medical therapy in axSpA, aimed to ameliorate physical disability as well as cardiorespiratory complications of the disease^46^. Thus, it is possible that physical exercise could have an influence on circulating CPT levels, lowering them, and hence possibly explaining our results. Unfortunately, we could not get enough information on the physical activity/therapy performed by our patients. Another potential explanation for the decreased levels of circulating CPT observed in our patients could be that they are reflecting an accumulation of CPT in the synovial fluid, based on the small size of CPT (36.5 kDa), which enables it to diffuse between inflamed tissues and circulation^47^. In this regard, previous reports have shown that CPT may diffuse from the inflamed joints into circulation^47,48^. Similarly, it has been suggested that inflammatory cells expressing CPT are activated and transmigrate from peripheral circulation, through the endothelium, to the inflamed tissues^34,43^. Thereby, as suggested by Levitova *et al*., decreased serum levels of CPT may mirror local inflammation^45^ (e.g. in synovial tissue). Even though axSpA is a predominantly axial disease, rarely presenting peripheral involvement, around 30% of our axSpA patients did exhibit peripheral arthritis/synovitis. Accordingly, it is possible that serum levels of CPT may be reflecting a certain degree of local inflammation in sacroiliac, costovertebral or facet joints, which are often affected in axSpA. In addition, it was reported that the levels of circulating CPT decrease after effective treatment in rheumatic diseases^31,49^. In the present study we assessed the influence of the different therapies that patients were receiving at the time of the study. At this time, the different modalities of treatment did not show any statistically significant effect on CPT serum levels. It is important to note that, in spite of the low activity of the disease displayed by our patients (based on the BASDAI value), they probably still have a certain degree of inflammation. Thereupon, another potential hypothesis to explain our results could be that this might be triggering compensatory mechanisms to reduce the detrimental effect of this remaining inflammation (by reducing, for example, the levels of pro-inflammatory molecules such as CPT, either in a direct or indirect fashion).

Regarding the role of CPT in CV disease, as above mentioned, in previous studies this molecule has been linked to atherosclerosis in different chronic inflammatory diseases^26–30^. Among them, the only study that included axSpA patients was one in which 12 AS patients were studied along with 15 rheumatoid arthritis and 9 psoriatic arthritis patients (as a single group). In such a study, it was reported that CPT associated with aortic stiffness, but not with cIMT values^30^. In our present study we did not disclose an association between CPT levels and cIMT values or presence of plaques, as surrogate markers of CV disease. However, when we assessed the potential link between CPT levels and metabolic risk factors, an association between CPT and lipid profile parameters emerged in our axSpA patients. In this regard, we found an inverse association between HDL-cholesterol and CPT in AS, and a positive correlation between this protein and the AI in both types of axSpA. These results on lipid association with CPT were further confirmed in our control cohort. Our results are in line with those obtained in previous studies, in which CPT negatively correlated with HDL-cholesterol in individuals with no previous history of CV disease and in obese patients^27,50,51^. This further supports the importance of CPT in the atherosclerotic process, regardless of the pathogenic context. Hence, CPT adds to a list of inflammatory markers that are associated with decreased HDL-cholesterol levels^52^. Low HDL-cholesterol levels and high AI are markers of inflammation and dyslipidemia, factors that further enhance the risk of developing atherosclerosis. However, whether this is a direct effect of CPT on lipid metabolism or an indirect effect mediated by the inflammatory status should be further evaluated.

In accordance with the pro-inflammatory role of CPT, in the whole group of axSpA we disclosed a positive association with the levels of CRP, a widely known biomarker of systemic inflammation, at the time of diagnosis of the disease. This was also confirmed in the subgroup of AS patients, supporting the data previously reported by others^27,36,44^. In a further analysis, we observed that axSpA patients with CRP levels at disease diagnosis higher than 3 mg/L, a CRP level considered representative of high vascular risk^53^, also showed higher serum CPT levels. However, in line with previous reports^41,42^, no association was observed between CPT and BASDAI at the time of the study. Similarly, no association was noted between CPT and ESR at the time of disease diagnosis, or ESR and CRP at the time of the study. Even if some authors have previously reported a correlation between CPT and/or ESR^36,41^, others did not find such association^42^. It is plausible to think that CRP and ESR levels at the time of the study could be under the influence of the medical treatment received, physical therapy and changes in lifestyle aimed to improve the quality of life of axSpA patients.

It is worth mentioning that, as occurs with other rheumatic diseases, the current tools used to predict the individual’s absolute risk for CV disease (such as lipid profile, CRP or other inflammatory markers) were found to underestimate the actual CV risk of patients with axSpA^12^. Consequently, the search for additional tools that may help to identify axSpA patients at high risk of CV events is of main importance. Thereby, we believe a combination of a series of biomarkers (among them CPT), rather than the assessment of a single one, along with the use of non-invasive surrogate markers, such as the carotid ultrasonography, may be needed to reach an adequate stratification of the actual CV risk in axSpA patients.

In summary, our study shows no relationship between CPT levels and markers of subclinical atherosclerosis in axSpA patients undergoing therapy who had low disease activity. Nevertheless, it demonstrates that CPT is associated with adverse lipid profiles and inflammatory markers in a large cohort of axSpA patients. This may be suggesting an indirect action of this molecule on the development of atherosclerosis. The fact that lower CPT serum levels were observed in our patients when compared to controls warrants further investigation to determine whether axSpA-intrinsic factors, specific characteristics of our patients or even the therapy used for the management of the disease could be implicated in the modulation of their circulating serum CPT levels, but not affecting its association with CRP or parameters linked to adverse lipid profile. Moreover, further molecular studies are warranted to elucidate the exact mechanisms by which CPT exerts its action in the development and progression of atherosclerotic disease in our patients. Additionally, it will be interesting to perform comparative studies on the association of CPT with subclinical atherosclerosis and metabolic risk factors in other inflammatory arthritides, such as psoriatic arthritis and rheumatoid arthritis.

## Methods

### Patients and controls

All the experiments involving humans and human blood samples were carried out in accordance with the approved guidelines and regulations, according to the Declaration of Helsinki. Furthermore, all experimental protocols were approved by the Ethics Committee of Clinical Research of Cantabria (CEIC-C, Number of reference 7/2016). Informed written consent was obtained from all subjects.

163 patients diagnosed with axSpA seen over a 3-year period at Hospital Universitario Marqués de Valdecilla and Hospital de Laredo (Cantabria, Spain) that fulfilled the ASAS classification criteria^54^ were recruited for this study. None of them had experienced CV events or had diabetes mellitus, chronic kidney disease, inflammatory bowel disease or psoriasis. 44 out of 163 patients fulfilled the definitions for nr-axSpA^54^, while the remaining 119 patients also fulfilled definitions for AS according to the 1984 modified New York criteria^55^. 63 healthy controls (who did not have history of CV events or chronic inflammatory diseases) were also recruited for the comparative analysis.

Data on sex, age, body mass index (BMI), blood pressure, total cholesterol, HDL- and low density lipoprotein (LDL)-cholesterol, and triglycerides at the time of study, and history of traditional CV risk factors (smoking, obesity, dyslipidemia and hypertension) were collected. Obesity was defined if BMI (calculated as weight in kilograms divided by height in squared meters) was >30. Patients were considered to have dyslipidemia if they had hypercholesterolemia and/or hypertriglyceridemia (defined as diagnosis of hypercholesterolemia or hypertriglyceridemia by the patients’ family physician, or total cholesterol and/or triglyceride levels in fasting plasma being >220 and >150 mg/dL, respectively). In those patients with total cholesterol between 200 and 220 mg/dL, a diagnosis of dyslipidemia was considered if the AI (total cholesterol/HDL-cholesterol) was ≥4.1. Patients were diagnosed as having hypertension if blood pressure was >140/90 mmHg or if they were taking antihypertensive agents. Information on CRP and ESR at the time of the study and at disease diagnosis was assessed, as well as information on disease activity at the time of the study (by calculating the BASDAI value) and therapy, including treatment with anti-TNF-α agents, DMARDs, NSAIDs and statins. The main demographic, laboratory and CV disease-related data of controls and patients are displayed in Table 1.

### Carotid ultrasonography

Carotid ultrasonography was performed in all the patients to assess the presence of abnormal cIMT values in the common carotid artery and the presence of focal plaques in the extracranial carotid tree (as surrogate markers of CV disease), as previously reported^10,11,13^.

### Study protocol

Determinations were made in the fasting state. Blood samples were taken for measurement of ESR (Westergren), CRP (latex immunoturbidimetry) and lipids (enzymatic colorimetry). Commercial enzyme-linked immunosorbent assay (ELISA) kits were used to measure serum CPT (HK325, Hycult Biotech, the Netherlands) according to the manufacturer’s instructions. All samples were analysed in duplicate.

### Statistical analysis

The analyses were first performed in the whole cohort of axSpA patients, and later patients were stratified into AS and nr-axSpA, since there is still concern on whether AS and nr-axSpA are distinct but overlapping disorders or two phases of the same disease^56^. Data were expressed as mean ± standard deviation (SD) and/or median [interquartile (IQ) range] for continuous variables, and number of individuals (n) and percentage (%) for categorical variables. Differences in CPT levels among the study groups were assessed by ANCOVA adjusting for potential confounding factors: sex, age at the time of the study and classic CV risk factors (smoking, obesity, dyslipidemia and hypertension). When comparing CPT levels between the two subtypes of axSpA we also performed an adjustment for ANCOVA using the above mentioned confounding factors along with HLA-B27 status. Correlation between CPT and continuous variables was performed via estimation of the Pearson partial correlation coefficient (*r*) adjusting for sex, age at the time of the study, classic CV risk factors and HLA-B27 status. Associations between categorical features and CPT concentrations were assessed by ANCOVA adjusting for sex, age at the time of the study, classic CV risk factors and HLA-B27 status. The correlation between CPT levels and continuous variables in healthy controls was performed via estimation of the Pearson partial correlation coefficient (*r*) adjusting for sex, age at the time of the study and classic CV risk factors.

Pearson partial correlation coefficients (r) were categorized as follows: 0.1 < |r| < 0.3 = small correlation; 0.3 < |r| < 0.5 = medium/moderate correlation; |r| > 0.5 = large/strong correlation. Two-sided *p* values ≤ 0.05 were considered to indicate statistical significance. Statistical analysis was performed using STATA® v. 11.1 (StataCorp, College Station, TX, USA).

## Data Availability

All data generated or analysed during this study are included in this published article.
